# P-1385. Improving Sexually Transmitted Infections Screening at a Quaternary Care Hospital Ryan White HIV Program: A Quality Improvement Initiative

**DOI:** 10.1093/ofid/ofae631.1561

**Published:** 2025-01-29

**Authors:** John Patrick Uy, Auden McClure, Antonia Altomare

**Affiliations:** Dartmouth Hitchcock Medical Center, Evansville, Indiana; Dartmouth Hitchcock Medical Center, Evansville, Indiana; Dartmouth-Hitchcock Medical Center, Lebanon, New Hampshire

## Abstract

**Background:**

Screening for sexually transmitted infections (STIs) such as gonorrhea, chlamydia, syphilis and hepatitis C (among men who have sex with men) are part of core performance measures and a reporting requirement for every Ryan White HIV/AIDS program. Dartmouth Health's Ryan White HIV program screening rates for these STIs as of August 2022 were ranging from 38% to 44%. The aim of this quality improvement initiative was to improve the screening rates to 80% by June 2024.

**Figure 1:**
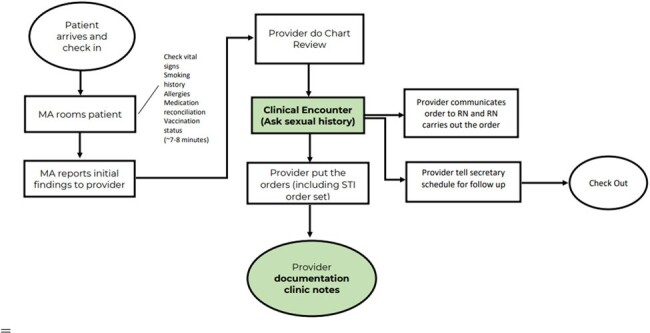
DH-Bedford Clinic Process Flow Map Highlighted in green are parts of the process where observation of clinical encounter and retrospective chart review were conducted.

**Methods:**

We used principles of models for improvement recommended by Institute of Healthcare Improvement (IHI), clinical microsystem approach and other quality improvement (QI) techniques in this project. The project had two phases. The initial phase comprised of an audit of the program through observation of clinical encounters, semi-structured interviews and retrospective chart review, while the implementation phase involved three quality interventions: (1) Presentation of initial chart audit findings and educational session about STI guidelines, (2) Use of STI screening cards (main intervention), (3) Practice feedback and regular updates. Analysis and display of data were presented using statistical control process (SPC) charts to identify any significant changes in the system or process

**Table 1:**
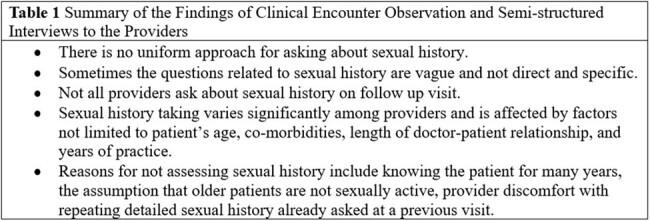
Summary of the Findings of Clinical Encounter Observation and Semi-structured Interviews to the Providers

**Results:**

From August 2022 to March 2024, we increased our screening rates for gonorrhea from 38% to 66%, chlamydia from 38% to 62%, syphilis from 44% to 64% and hepatitis C (among MSM) from 39% to 82%. Several favorable special cause variations were observed in all outcome measures after the implementation phase.

**Figure 2:**
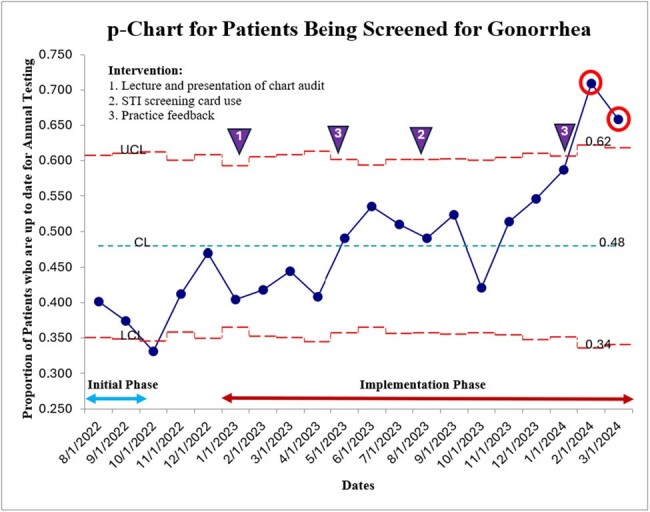
p chart for Patients Being Screened for Gonorrhea

This figure illustrates the proportion of patients seen in clinic each month who are screened for gonorrhea at least annually. The mean screening rates is 48%. The upper control limit is 62% while the lower control limit is 34%. There are two special cause variations (2 data points outside the UCL - red circle).

**Conclusion:**

The use of quality improvement principles and methods to routinize sexual history taking can improve screening rates for sexually transmitted infections among adult patients in a Ryan White HIV program. Incorporation of the questions through the EMR is the next step for it to become more sustainable.

**Figure 3:**
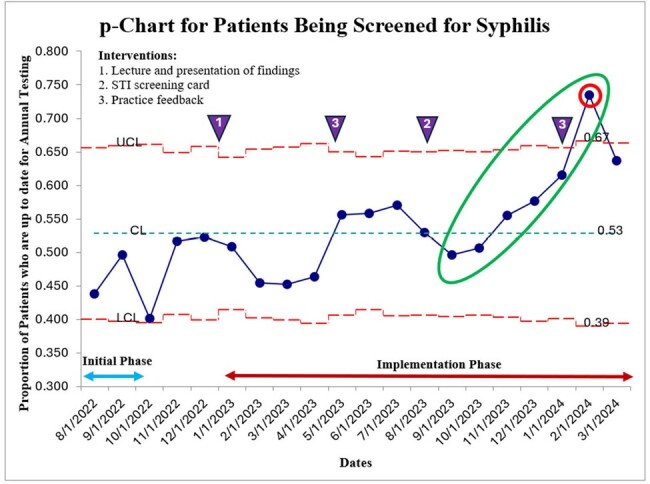
p chart for Patients Being Screened for Syphilis

This figure illustrates the proportion of patients seen in clinic each month who are screened for syphilis at least annually. The mean screening rates is 53%. The upper control limit is 67% while the lower control limit is 39%. There are two special cause variations. First is upward trend (green circle) from 8/2023 to 2/2024. The second is a point outside the UCL in 2/2023.

**Disclosures:**

**All Authors**: No reported disclosures

